# From Grief to Grievance: Combined Axes of Personal and Collective Grief Among Black Americans

**DOI:** 10.3389/fpsyt.2022.850994

**Published:** 2022-04-28

**Authors:** Da'Mere T. Wilson, Mary-Frances O'Connor

**Affiliations:** Department of Psychology, University of Arizona, Tucson, AZ, United States

**Keywords:** grief, Black/African American, collective grief, loss, racism

## Abstract

In the current article, we argue that the current conceptualization of grief as “the acute pain that accompanies the loss of a loved one” is too narrow in scope. Specifically, our current conceptualization of grief fails to account for the various ways in which grief is manifested amongst Black Americans. Throughout the article, we explore how the history of the racialization of Black people in America has resulted in a unique experience of loss, grief, and bereavement which previous research has largely failed to elucidate. Additionally, we explore how grief catalyzes political and social action. The article also proposes a novel theoretical conceptualization of personal and collective grief to deepen our conceptualization of grief amongst Black Americans. Finally, we posit that we must also consider how to further research on this collective grief to increase our understanding of it and to account for similar phenomena that may exist in communities who've had similar experiences (e.g., Indigenous peoples in the Americas and Dalits in India).

## Introduction

In the summer of 2015, 7 days after Sandra Bland was found asphyxiated in her jail cell after being arrested for not using her turn signal, YouTube creator “Evelyn from the Internets” posted a video entitled “Calling in Black” in which she describes the process of waking up to the news of the unjust murder or assault of *another* Black person. She imagines a world in which it is socially acceptable to call in Black to work as one would call in sick (1). In this video, Evelyn utilizes humor to highlight the absurdity of the repetitious violence to which Black people are exposed to whilst, simultaneously, regular life drones on in the background. When describing the various reactions an individual Black person may have to this quotidian violence, Evelyn states “…the other day when I was driving to work and I noticed water randomly pouring from my eyes, I realized something…I was grieving.” With over five million views across social media platforms, this skit seems to have tapped into a very relatable experience for Black Americans, and, as we will argue throughout this article , the video explicates a key feature of this experience: grief.

The current conceptualization of grief as “the acute pain that accompanies the loss of loved one” is too narrow in scope to encompass Black grief (2). Specifically, current theoretical conceptualizations of grief fail to account for the various ways in which grief is manifested amongst Black Americans. The racialization of Black people in America has been marked by enslavement, historical and ongoing racial violence, historical and ongoing economic and land dispossession, pervasive structural inequality, and staggering amounts of loss. We must expand our definition of grief to account for the pain that occurs within the Black community after the loss of a loved one, the loss of land, the loss of a sense of safety, and the loss of members of the community due to direct and indirect acts of racist violence. Additionally, we must expand the scope of grief research to consider the experiences of Black Americans through a multisystemic lens that adequately contends with the historical, socio-political, economic, and psychological components that are critical to understanding this unique experience of grief. This article reviews and characterizes the unique and understudied experience of grief for Black Americans, including the unequal distribution of grief, vulnerability to premature death, and historic and ongoing violence. We argue that grief for Black Americans is both different in sheer quantity (because mortality rates are disparate) and qualitatively different (because collective grief is an unstudied but defining feature). This article creates a framework to consider these two axes of grief among Black Americans (i.e., personal and collective grief) and discusses how the inseparable nature of these axes, experienced as a singular phenomenon, can be the motivation for expressing justifiable grievance. We send a call to action for further research to advance the clinical science understanding of collective grief. This may have the future benefit of also increasing our understanding and accounting for similar phenomena in other communities with similar experiences (e.g., Indigenous peoples in the Americas and Dalits in India).

## Grief Is Understudied in Black Americans

Grief will always be prolonged, as long as injustice is prolonged.

—Tashel Bordere, Ph.D.

Grief is typically defined as a primarily emotional reaction to the loss of a loved one through death. It also incorporates diverse psychological (cognitive, social-behavioral) and physical (physiological-somatic) manifestations (2). The loss of a loved one is consistently ranked as one of the most stressful life experiences (3). Cognitive manifestations of grief can include disbelief, yearning, rumination, or difficulty concentrating. Emotional manifestations of grief can include sadness, longing, loneliness, anxiety, sorrow, guilt, anger, and sometimes relief. Physical manifestations of grief can include shortness of breath, feelings of emptiness, muscle weakness, tension, pain, and changes in appetite and sleep (4).

The aforementioned conceptualizations of grief are all individual processes. However, grief can also be experienced communally. Collective grief occurs when a community collectively experiences an extreme change or loss (5). Eisenbruch points out that Western psychiatry has conventionally considered the individual to be at risk, but that attention could usefully be shifted from individuals to an entire uprooted people. In a recent critique of grief research that occurs almost exclusively in the Global North, Stelzer et al. write, “Many cultural factors, working invisibly in the background, affect the way symptoms are expressed, perceived, assessed, interpreted, and documented” (6).

Since the early twentieth century, heavily influenced by Sigmund Freud's “Mourning and Melancholia” (7) essay, the phenomenon of grief has become a topic of study within the field of psychology. Researchers have elucidated many key findings about the common symptoms, coping mechanisms and the mental and physical health outcomes of grief (8). However, Black Americans have largely been underrepresented within research on understanding the psychological impact of grief and bereavement. One recent review found that of the ~4,000 articles published on grief and bereavement, only ~100 of these studies included Black Americans in the sample (9).

Within the few dozen studies that have used exclusively Black Americans samples, most have focused on homicide related loss. While it is true that African Americans are more likely to experience the murder of a loved one, the hyper focus on homicide loss has led to a dearth of research on how other types of loss affect this community (9). However, it is important to note that researchers have found that losses that occur without warning or in a traumatic or violent manner (e.g., homicide or suicide), may result in what has been coined traumatic bereavement. Individuals who experience traumatic bereavement must both mourn the loss and cope with the trauma that accompanied the death. Thus, those who experience traumatic bereavement typically experience more intense and severe grief and trauma related symptomology when compared to those who experience a natural death of a loved one (10). A few studies, on the impact of traumatic loss on Black people bereaved by homicide, have found increased rates of PTSD, and “complicated grief” amongst this population and these symptoms may be exacerbated by lack of access to psychosocial services (11, 12). However, these studies were based on relatively small samples and further research is needed to further explicate the true nature of this phenomenon when considering the impact of structural racism.

This lack of representation, plus the prevalent universalist research framework in psychology, has led to a lack of understanding of how the specific contextual factors of living as a Black person, in a Western country like the United States, may differentially impact the experience of grief. Universalism within psychology refers to the idea that the range of human experiences, from basic core needs and psychological processes to core values, are innate and therefore similar across peoples and cultures (13). Although research has shown that a person's ethnicity, social factors, and the geographical and historical context will influence how a person grieves, most psychological research, as Granek notes, “continues to assume that all people can be compared to a universal norm that has historically been based on White, European men” (14). One recent and particularly troubling example of this can be seen in the research supporting the recent inclusion of Prolonged Grief Disorder in the ICD-11. Within the study tasked with the psychometric validation of Prolonged Grief Disorder for the DSM-5 and ICD-11, the sample used data from the Yale Bereavement Study (YBS) which was stated to be 95.3% white. The researchers included no other information about the ethnicity breakdown of the other 4.7% of the sample (15). Despite this overwhelmingly uniform sample, the discussion in the article states “…the YBS sample was 73.7% female compared with 80.7% of the US widowed population and 95.3% white compared with 80.2% of the US widowed population.” Despite this glaring difference in the sample and the population, they later concluded the results generalize to most bereaved individuals. However, this conclusion is erroneous. The article notes that the sample is similar to the US population of widows, but the 15% disparity from 80 to 95% in the study sample is one of note. Additionally, the generalized conclusion that PGD is psychometrically valid despite this uniform sample is an example of the prevalence of the universalist framework in psychological science.

One study found that African Americans show a higher prevalence of prolonged grief disorder when compared to white Americans (16). This study also used the same YBS data, and concluded that African Americans have twice the rate of PGD prevalence when compared to whites. These conclusions also stand on shaky ground as these results are based on the assessment of only 66 African American participants (compared to 471 white participants). Additionally, the article further perpetuates the pathologization of Black American experiences and does not critically analyze the lack of representative validation of PGD. The model of decontextualized comparison methodologies combined with the pathologization of grief experiences has been replicated in many studies attempting to understand the Black grief experience (17, 18) and have further contributed to the view of Black Americans as deficient and dysfunctional.

Seeking to understand the experience of grief and its impact on psychological wellbeing is a noble pursuit within the field. However, thus far, a preponderance of the research on grief and bereavement has included samples which are mostly white, middle-aged, middle- or upper-class people. Research that has included Black populations has not adequately contended with the unique contextual factors including culture, racialization, and other factors which shape their grief. There is a great need for more studies that both include more Black Americans in the sample, and all other ethnicities, and specifically assess how ecological systems and cultural factors may influence the specific manifestations of the Black American grief experience. For example, during chattel slavery, enslaved Black people were routinely forced to separate from their spouses, children, and family. These separations were often violent in nature and are generally mentioned when discussing the trauma associated with enslavement (19). However, often overlooked is the grief that accompanies these traumatic losses as well as the reverberating effects across generations. In Solomon Northup's accounts of his time enslaved, he detailed the grief of a woman, Eliza, who had been separated from multiple children. When describing Eliza's separation from her daughter Emily, Northup notes “never have I seen such an exhibition of intense, unmeasured, and unbounded grief” (20). These injuries perdure in the present day as Black families continue to be upended by disproportionate incarceration (21), and disproportionate “child welfare” separations (22). While the experience of death, grief, and loss are universal, the ways in which an individual conceptualizes and subsequently responds to loss are heavily mediated by various social factors, some of which will be explored in the next section.

## Grief Is Not Equally Distributed

Racism…is the state-sanctioned or extralegal production and exploitation of group-differentiated vulnerability to premature death.

—Ruth Wilson Gilmore, *Golden Gulag*

Disproportionate experiences with grief, loss, and bereavement due to the overlapping and interrelated forces of systemic discrimination and oppression is a defining characteristic of the Black experience in America. The first axis of this experience could be labeled personal grief. According to the 2020 National Vital Statistics report, the average life expectancy for a white American is 78 years and the average life expectancy for a Black American is 72 years; this life span gap increased from the previous year as the average life expectancy for Black American's decreased by 2.7 years due in large part to the unequal distribution of COVID-19 deaths (23). The COVID-19 pandemic death rate for Black Americans is 1.7 × higher than white Americans (24), and consequently, Black children were 2.4 times more likely than white children to lose a parent or caregiver (25). In addition to living much shorter lives, Black Americans often live sicker lives. Black American's are twice as likely to die from heart disease than whites, 50% more likely to have high blood pressure, and are more likely to die at early ages from all causes, according to the CDC (26).

However, the consequences of bereavement on surviving grieving family members are a largely overlooked area of racial disadvantage. The statistics above mean that Black Americans have both exposure to deaths of younger family members and friends, and also increased numbers of deaths in their lives. According to a 2017 study, Black Americans are significantly more likely than whites to have experienced the death of a mother, a father, and a sibling from childhood through midlife (27). Before the age of 10, Black children are three times more likely to lose a mother. From young adulthood through later life, Black people are also four times more likely than whites to have experienced the death of a child and twice as likely to experience the death of a spouse (27). The death of a loved one is widely documented as a significant stressor that undermines health and the repeated and early life course exposure to death is unique to Black Americans and has lifelong consequences for relationships and health (28).

Bereavement is a health disparity; these multiple losses have medical consequences. In epidemiological research by Lewis et al., Black women in mid-life who experienced three or more upsetting deaths across 12 years of assessment had greater carotid intima media thickness, a marker of cardiovascular risk measured by ultrasound (29). In addition, although being widowed is associated with significantly higher odds of subsequent death of the surviving spouse across all racial-ethnic groups (e.g., the widowhood effect), the mortality difference by widowhood status is 1.5 times greater among Black Americans (30).

Interpretations of health/death disparity data are often blamed on Black Americans. According to the 2017 “CDC African American Health Report”, social factors including higher unemployment rates, higher poverty rates, low home ownership rates, and high obesity rates are to blame for these health disparities. This report neglects to mention the underlying causes of all of the mentioned social factors—racialization and racism. The CDC points to high unemployment rates without including the relevant data on chronically underfunded and under-resourced education systems in Black neighborhoods, or the role of the school to prison pipeline and mass incarceration (21). High obesity rates are incomplete information without including the lack of grocery stores that plague Black neighborhoods, or the fact that Black farmers lost 93% of their land from 1910 to 1997 through racist land dispossession (31, 32). The model of blaming the individual for the manifestations of systemic racism is chronic also within psychological science. In a recent call to action, Volpe et al. (33) noted that psychological science has taken a traditionally ahistorical, acontextual, risk-based, and individual approach to defining and examining concepts of race, racism, and health disparities. This deficit-based individual-level approach implies that psychological science can intervene on racial health disparities by imparting the adequate and appropriate skills and psychoeducation to buffer risk upon Black individuals and communities. However, this framework fails to address the systemic racism that undergirds these observed health disparities or, as Ruth Wilson Gilmore writes in the quotation above: vulnerabilities to premature death.

## Ongoing Violence

How might we understand mourning, when the event has yet to end? When the injuries not only perdure, but are inflicted anew? Can one mourn what has yet ceased happening?

—Sadiya Hartman, *The Time of Slavery*

While Black Americans are contending with living shorter and sicker lives due to the material manifestations of systemic racism, they are simultaneously exposed to the abuse and death of fellow Black Americans due to racist violence. This chronic exposure to racist violence marks the second axis of grief that is characteristic of the Black American experience—collective grief.

Everyday life is marked by interludes of quotidian racial violence for the average Black American. Whether it be directly experienced (e.g., having a racial slur yelled at you) or indirectly experienced (e.g., waking up to the news of *another* unarmed Black person murdered by police), this commonplace violence is reflective of the normality of racism in the US. These grotesque interludes of violence have been characteristic of the Black American experience since the colonial period. Enslaved Africans were routinely made to witness the horrible acts of violence being done upon fellow slaves. During the Jim Crow era, Black people were routinely brutalized and hung in the public square. In the modern era, social media has introduced a new scale of witnessing to which Black people are subjected. The violence is not limited to local social spheres, but now millions of people can be informed of the latest brutal act of racist violence within minutes (34).

Parasocial grieving is most often used to describe the experience of loss that develops between media users and media personae. Parasocial relationships are one-sided, in which individuals develop an attachment to someone they do not have a personal relationship with, such as a celebrity, a character in a TV show, or an internet personality. Parasocial relationships were first explored in the mid-twentieth century, when Horton and Wohl (35) argued that radio listeners seem to develop intimate bonds with radio personalities that they often listened too. These relationships resemble interpersonal relationships, and prior to social media, parasocial grief was experienced primarily individually or introspectively. However, as discussed by Sanderson and Hope Cheong (36), new media technologies have created the opportunity for individuals to have access to vast social networks consisting of connections across the globe. For example, after the death of singer Michael Jackson, fans were not experiencing the loss of their parasocial attachment alone, but were actively mourning and finding others who felt the same *via* social media networks (36). Thus, social media has created a unique grieving space in which people can connect with others experiencing the same loss across the world.

Previous models of parasocial grief contend a strong attachment prior to the death of a celebrity will lead to feelings of loss or grief (37). However, we posit that the same perceived relationship could be facilitated by intense media coverage of the death of a layperson, especially if there is a connection to the deceased through shared race, gender, or other identity. For example, media exposure to high profile instances of police killings might lead to parasocial grief, especially for Black Americans. The groundwork for intense experiences of parasocial grief, facilitated by new media technologies, was laid throughout the history of racialization of Black people specifically in the U.S context. To understand how parasocial relationships manifest within this community it is important to understand two key features of Black American ethnoracial identity: linked fate and fictive kinship.

Fictive kinship within the Black American community can be traced to the legacy of chattel slavery in the U.S. Fictive kinship can be defined as the extension of kinship or familial obligations and relationships to individuals not otherwise related by blood or marriage (38). Gutman's s classic 1976 work provides vast historical evidence of the practice of establishing fictive kinship ties amongst enslaved Africans in the U.S (39). Guttman revealed that enslaved parents and other adults taught children to address older persons who were unrelated to them by either blood or marriage as “Aunt” or “Uncle”. Additionally, due to the nature of family separation and natal alienation during chattel slavery, many enslaved African “replaced” their absent extended family with “fictive kin” with whom they were surrounded. Contemporarily, the family structure of many Black American families often includes non-familial fictive ties. These relationships serve to enhance social control, broaden mutual support networks, and create community (38). Because of this feeling of familial relatedness other Black people, created out of necessity, parasocial grief may be more likely to occur in the Black community following the death of a person who could be family.

In addition, as defined by Dawson (40), linked fate is the recognition that individual life circumstances are inextricably tied to the race as a whole. Linked fate is a feature of ethnoracial identification which begins with a feeling of closeness to others who identify with a group label and evolves into the acceptance of the idea that the individual's life chances are linked to the group. In other words, linked fate is the ever-present awareness that what happens to the group will also affect the individual member. This linkage may also increase the likelihood of parasocial grief within the Black community when a previously unknown Black person is brutally attacked or murdered, because of the feeling that this could be the fate of oneself, or one's brother, son, or father.

Ethnoracial identity has been long considered a protective factor for mental health among Black Americans. However, recent research has suggested that this identification may in fact be a “double edged sword” (i.e., while ethnoracial identification has many positive and protective effects on health, it also has some deleterious effects) (41). One of the ways in which ethnoracial identification can be problematic is through the phenomenon of linked fate. Black Americans disproportionate exposure to the death of a loved one, coupled with the highly visible and broadly reported deaths of Black Americans coalesce to create an overarching sense of threat and distress, especially amongst those whose ethnoracial identification is most salient. While feelings of linked fate can be a powerful source of group mobilization, recent studies have shown that they are also linked to negative mental health effects [see (41, 42)]. As noted by Monk (42), it is key to understand that while a sense of linked fate may have costs to the individual, these costs (e.g., anxiety and anger) may be the fuel to political mobilization.

Linked fate and fictive kinship are two important features of Black American racial identity. Both these phenomena contribute to a greater sense of community interconnectedness *via* shared racial/ethnic identity. This community interconnectedness and racial identification plays a protective role said to moderate the relationship between discriminatory experiences and psychological wellbeing (43–45). These identity markers developed as the result of various aspects of Black racialization in the U.S and have shifted form as social media has created ever larger social networks. However, while these phenomena may have protective elements, their manifestation within the modern world may prove to be a double-edged sword for those whose race/ethnicity is more central to their identity. As Black Americans are already more apt to seeing strangers as close kin and sense their fate is linked to what happens to other Black people, they may be more likely to form parasocial relationships and by extension experience parasocial grief in the digital landscape. Parasocial grief that develops as a result of media exposure to acts like police brutality may, therefore, represent a unique mechanism through which the social stress of discriminatory treatment could exert wide-reaching impacts on the population.

## Grievance

To be a Negro in this country and to be relatively conscious is to be in a rage almost all the time.

—James Baldwin, *The Negro in American Culture*

While grief is usually conceptualized within the context of loss, the experience is often very generative for the individual or community that has experienced the loss. Perhaps the best way to understand the generative nature of grief is through the word's etymological roots. The word “grief” stems from the Old French *grever* meaning afflict, burden, or oppress, which became the Old French *grief* meaning wrong, grievance, injustice, or calamity. Individuals who experience the loss of a loved one often note experiencing anger because of the acute sense of unfairness or injustice that accompanies the loss of a loved one (46).

Emotions include a signaling function. Anger, specifically, is a natural response to perceived intentional injury, mistreatment, or victimization. It signals the need to increase activity and mobilize efforts to defend oneself or a loved one. The anger and sense of injustice that marks the grief experience makes it a powerful experience with the potential to catalyze efforts to “defend oneself or loved ones” against injustice. This act of resistance and thriving in the face of grief is functional and has both individual- and community-level assets (33).

The catalyzing nature of anger in grief is most readily observed within modern social justice movements. In 1963, four young Black girls were murdered in the bombing of the Birmingham, Alabama 16th Street Baptist Church orchestrated by the local Ku Klux Klan. In his eulogy at the funeral service for the girls, Dr. Martin Luther King, Jr. proclaimed, “… in a real sense they have something to say to each of us in their death…”. They say to each of us, Black and white alike, that we must substitute courage for caution. They say to us that we must be concerned not merely about who murdered them, but about the system, the way of life, the philosophy which produced the murderers. Their death says to us that we must work passionately and unrelentingly for the realization of the American dream (47). This call to action was preceded by days of protests at the scene of the bombing, which resulted in the death of two more young Black men killed by the national guard. In his eulogy, Dr. King formalized the grief to grievance process by calling for community members to use the agony from this ultimate injustice, the murder of innocent children, to fuel the continued struggle for human rights. The outrage and collective grief over the death of the four young girls helped to build increased support, particularly from white moderates, of the civil rights movement and desegregation movements. The galvanizing energy from the grief, felt by the nation, for these four girls spurred the reenergized movement that led to the passage of the Civil Rights Act within the next year (1964) and the Voting Rights Act in 1965.

This activating aspect of grief was also on display in the summer of 2020 after the murder of George Floyd in Minneapolis, MN. For 8 min and 46 s, the world watched as Floyd begged for mercy as his murderer held his knee to his neck. Fueled by mass sharing *via* social media, the video of George Floyd's murder was shared and reshared over and over again under the notion of “raising awareness”. This collective witnessing, amongst other things, resulted in the largest protest movement in the history of the United States. However, the casual ways in which people consumed his death, and Black death in general, may elicit grief (in addition to trauma and other psychological consequences) in Black people. The outrage over the injustice of Floyd's murder sent shockwaves throughout the world and brought between 15 and 25 million people out into the streets, making the George Floyd protests the largest in U.S history (48). However, these protests were not just displays of anger but also a space of collective mourning and as Judith Butler notes, “…open grieving is bound up with outrage, and outrage in the face of injustice or indeed of unbearable loss has enormous political potential (49).” The George Floyd protests lived up to what Butler (49) describes; political potential and an activated racial reckoning that reverberated throughout all aspects of life in the U.S. Major corporations began to proclaim #BlackLivesMatter, the Minneapolis police department was defunded, and the subsequent conviction of the officer who murdered George Floyd catalyzed countless regular individuals to confront the myth of a post-racial America and commit to fighting for racial justice within their own spheres of influence.

## Discussion

The recent pandemic has only amplified bereavement as a health disparity. The consequences of bereavement on surviving grieving family members are largely overlooked in the study of racial disadvantage, and collective grief that includes unique aspects for the Black community, including fictive kin and linked fate, is understudied. The combined mantle of personal and collective grief that Black Americans experience is unrelenting.

As the field of grief and bereavement research continues to grow, we must reckon with the idea that grief exists outside of the accepted definition of “the emotional reaction to the loss of a loved one” in order to incorporate collective and historic grief. We must contend with the myriad ways that grief exists outside of this individualistic, white, western frame. Black people have been traditionally underrepresented in the grief and bereavement research literature. This lack of representation, coupled with a lack of consideration of the unique socio-cultural contextual history of Black people in the west, has left a dearth of understanding of the Black experience of grief. Further, universalist approaches to psychology have led to a further pathologization of Black American grief experiences. Grief research has also failed to interrogate how the disproportionate distribution of bereavement, earlier and more often throughout the life course, might differentially impact Black bereaved people.

By centering our understanding on this more variegated grief, we may find the ways in which it is a strength and therefore change psychological science's Black health narrative. As racialization, and racism (both interpersonal and structural), continues to be an ongoing reality of society, so does the fight for the liberation of oppressed people, including Black Americans. The galvanizing nature of grief is generally showcased on a collective level through things like public memorials or protests in situations of injustice. As the intertwined experience of personal and collective grief catalyzes collective action, which has positive consequences in the sphere of freedom making, it is also important to understand the potentially beneficial and deleterious effects of this phenomenon on the individual level.

**Figure 1:**
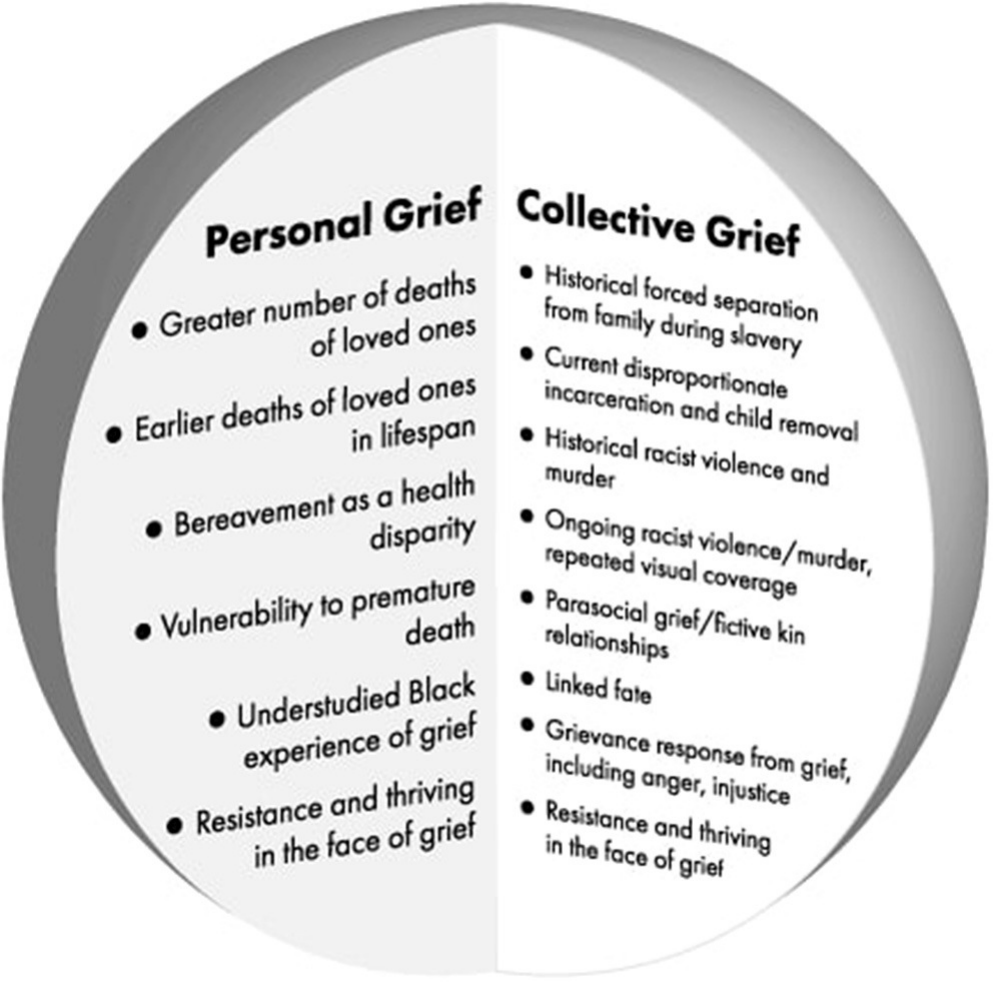
Examples constituting personal and collective grief axes of Black Americans.

Our conceptualization that grief for Black Americans, experienced as an inseparable personal and collective sense, is shown in Figure 1. This unique meshing of the personal and collective can catalyze righteous grievance, and this novel conceptualization is largely missing from the literature in grief research today. The potential far-reaching effects of the quantitatively and qualitatively different experience of grief on Black mental and physical health make this an all too important area for future research and analysis.

## Data Availability Statement

The original contributions presented in the study are included in the article/supplementary material, further inquiries can be directed to the corresponding author.

## Author Contributions

DW contributed to the conception and design of theory and brief review of the literature. DW wrote most of the manuscript with M-FO'C contributing to some sections. Both authors contributed to manuscript revision, read, and approved the submitted version.

## Funding

This material is based upon work supported by the National Science Foundation Graduate Research Fellowship under Grant No. DGE-1746060.

## Conflict of Interest

The authors declare that the research was conducted in the absence of any commercial or financial relationships that could be construed as a potential conflict of interest.

## Publisher's Note

All claims expressed in this article are solely those of the authors and do not necessarily represent those of their affiliated organizations, or those of the publisher, the editors and the reviewers. Any product that may be evaluated in this article, or claim that may be made by its manufacturer, is not guaranteed or endorsed by the publisher.
